# Effect of *Thymus vulgaris* L. essential oil and thymol on the microbiological properties of meat and meat products: A review

**DOI:** 10.1016/j.heliyon.2022.e10812

**Published:** 2022-09-30

**Authors:** Miklós Posgay, Babett Greff, Viktória Kapcsándi, Erika Lakatos

**Affiliations:** Department of Food Science, Faculty of Agricultural and Food Sciences, Széchenyi István University, 15-17 Lucsony Street, 9200 Mosonmagyaróvár, Hungary

**Keywords:** Thyme essential oil, Thymol, Antimicrobial effect, Meat product, Bio-preservative

## Abstract

Since foodborne diseases are often considered as one of the biggest public health threats worldwide, effective preservation strategies are needed to inhibit the growth of undesirable microorganisms in food commodities. Up to now, several techniques have been adopted for the production of safe and high-quality products. Although the traditional methods can improve the reliability, safety, and shelf-life of food, some of them cannot be applied without rising health concerns. Thereby, the addition of various phytochemicals has gained much attention during the last decades, especially for meat products that may be contaminated with pathogenic and spoilage organisms.

Thyme (*Thymus vulgaris* L.), as an important medicinal and culinary herb, is a promising source of bioactive compounds that have a great impact on the microbiological stability of meat by suppressing the undesirable microflora. However, the use of these antimicrobials is still facing difficulties due to their aromatic properties and variable efficacy against targeted species.

In this paper, we provide an overview on the potential effects of thyme essential oil (EO) and thymol as bio-preservative agents in meat products. Furthermore, this paper provides insights into the limitations and current challenges of the addition of EOs and their constituents to meat commodities and suggests viable solutions that can improve the applicability of these phytochemicals.

## 1 Introduction

As a consequence of globalization and active food trade, foodborne diseases caused by bacteria, fungi, viruses, and parasites have become one of the leading health problems worldwide (Lee and Yoon, 2021; Oliveira et al., 2019). Although every country has specific disease control and food safety regulations, the number of foodborne outbreaks has been continuously increasing resulting in product recalls and serious economic losses (Horn and Bhunia, 2018; Lee and Yoon, 2021). In general, the consumption of contaminated food products has been causing approximately 600 million cases of foodborne infections and 420,000 deaths annually (WHO, 2020). The microorganisms associated with food safety hazards mostly induce self-limiting diseases with symptoms of nausea, vomiting, abdominal cramps, diarrhea, and headache only (Lee and Yoon, 2021; Rivera et al., 2018), but foodborne illnesses can lead to chronic sequelae and even death in the case of highly susceptible groups of the population (i.e., elderly people, children under five, immunocompromised individuals, etc.) (Lund, 2019; Rivera et al., 2018).

Meat is one of the most perishable foodstuffs due to its susceptibility to microbial and oxidative spoilage (Kanatt et al., 2008; Tornuk et al., 2015; Wang et al., 2022b). Raw meat itself is often associated with various bacteria including *Salmonella*, *Campylobacter*, and *Escherichia coli* (Hennekinne et al., 2015), but commodities derived from pork meat, turkey, and broiler are also recognized as important sources of salmonellosis and other foodborne diseases (Boskovic et al., 2017; Cavadini et al., 1998; Hu et al., 2018).

For instance, many Shiga toxin-producing *Escherichia coli* related outbreaks were reported in the past (Chien et al., 2016). In 1999, more than 140 cases of foodborne illnesses were linked to the consumption of dry Hungarian and Cervelat salami contaminated with *Escherichia coli* O157:H7 (Graumann and Holley, 2008). Furthermore, 279 confirmed cases of *Escherichia coli* O157 infection were recorded in Scotland, after the consumption of meats supplied by a single food premise (Cowden, 1997; Cowden et al., 2001; Pennington, 2014). Meanwhile, the Jack in the Box incident in 1993 has remained one of the largest and deadliest cases of *Escherichia coli* O157:H7 outbreaks associated with restaurants: the consumption of rare hamburger patties sickened more than 700 people and killed four children (Pennington, 2010; Seo et al., 2014).

In addition to bacteria, fungi including *Penicillium*, *Aspergillus*, *Fusarium*, and *Alternaria species* can also contaminate various food commodities and secrete toxic secondary metabolites like mycotoxins (Medina-Córdova et al., 2018; Misra et al., 2018; Oyedeji et al., 2021). *Aspergillus* spp. and *Penicillium* spp. have been previously isolated from dry-cured meat, dry-smoked meat products, and canned poultry and beef (Odeyemi et al., 2020). *Aspergillus candidus* was detected on the surface of salami, other processed meat products, and dried fish (Hocking, 2006). Furthermore, *Cladosporium* spp. has been associated with various meat products including hams and sausages (Mizakova et al., 2002).

To mitigate the risk of foodborne pathogen contaminations, advanced processing and preserving techniques have been employed in the food industry (Horn and Bhunia, 2018). Despite the beneficial effects of artificial additives on food properties and deterioration, the perception of consumers towards processed food has become generally negative as they prefer no additives over synthetic chemicals. Meanwhile, products with the word “natural” on their labels and organically produced foods are often associated with a healthier lifestyle and are more desirable to consumers than their non-natural analogs (Barcenilla et al., 2022; Moreira et al., 2005; Perito et al., 2020). Owing to this recent trend of green consumerism and the unceasingly growing demand for safe and healthy meat products, novel preservation techniques, such as bio-preservatives derived from plants, animals tissues and products, or microorganisms, have been proposed and investigated extensively (Barcenilla et al., 2022; Quesada et al., 2016).

Although medicinal and aromatic plants and their essential oils (EOs) have been widely utilized for food preparation since ancient times (Giannenas et al., 2020), their role in bio-preservation has gained much attention in the past years only. Among these plants, thyme (*Thymus vulgaris* L.) proved to be a promising source of bioactive substances (Gedikoğlu, 2022) including EO and thymol which can prevent the growth and spread of undesirable microorganisms. However, their antimicrobial mechanisms at the molecular and cellular levels are still not fully understood and only a few EO-based, commercially manufactured food preservatives are available today (Oliveira et al., 2020; Pandey et al., 2021). In the case of fermented products, the potential effect of EO on beneficial microorganisms, such as mesophilic starter lactic acid bacteria (LAB), should also be taken into consideration (de Carvalho et al., 2015). Therefore, their practical application in meat products is still limited and requires detailed information regarding their properties (e.g., solubility, hydrophobicity, range of target microorganisms, minimum inhibitory concentration against specific microbes, mode of action, possible interactions with the food matrix, sensory changes, etc.) (Asensio et al., 2017; da Silva et al., 2021; Hyldgaard et al., 2012; Nowak et al., 2012).

For this purpose, this review summarizes and evaluates the findings of current scientific literature on this topic and suggests novel methods that can reduce the detrimental effects of these natural antimicrobials on meat quality.

## 2 Potency of medicinal and aromatic plants as preservatives for meat and meat products

Meat, the skeletal muscle of animals with any attached fat or connective tissues and offal excluding the bone and bone marrow, is a valuable source of protein, vitamins, essential fatty acids, and minerals (Bantawa et al., 2018; Williams, 2007). Although the meat of healthy animals contains almost no microorganisms, it is a highly perishable commodity (Bantawa et al., 2018) that provides an ideal environment for the growth of both spoilage and pathogenic bacteria such as *Acinetobacter* spp., *Enterobacter* spp., *Proteus* spp., *Lactobacillus* spp., *Pseudomonas* spp., *Leuconostoc* spp., *Yersinia enterocolitica*, *Salmonella* spp., *Listeria monocytogenes, Staphylococcus aureus, Clostridium botulinum, Escherichia coli, Campylobacter jejuni*, and *Clostridium perfringens* (Boskovic et al., 2015; Favaro and Todorov, 2017). These microbes may contaminate the fresh meat at different stages of the production via air, soil, water, gastrointestinal tracts, feces, lymph nodes, hide, processing equipment, or employees causing serious safety and quality issues in the meat industry (Abdel-Sater et al., 2017; Favaro and Todorov, 2017). In addition to the primary sources, re-contamination and cross-contamination can occur in domestic environments during improper food handling and food preparation practices (Boskovic et al., 2017).

To improve the microbiological safety and quality of meat and meat-derived products, several chemical, physical, and biological methods have been adopted (Delmore et al., 2000; Yu et al., 2021). Although the conventional meat preserving techniques such as dehydration, heat processing, smoking, curing, and low-temperature preservation have been successfully utilized for centuries, these technologies may be challenged by various factors (e.g., the composition of meat, requirement for large-scale equipment and higher costs, reduced efficiency, less durability, etc.) (Misra and Jo, 2017; Ren et al., 2021; Tajkarimi et al., 2010; ur Rahman et al., 2018).

On the other hand, the addition of chemicals or bio-preservatives is a more practical approach (Ren et al., 2021). Synthetic compounds, including antimicrobials, antioxidants, and anti-enzymatic preservatives, can improve the microbial shelf-life of meat products without drastically changing the texture, flavor, or color. However, consumers have expressed their concerns about the application of artificial chemicals because of their potential deleterious health effects (Anand and Sati, 2013; Pesavento et al., 2015; Roobab et al., 2021; Yu et al., 2021). For instance, nitrites and nitrates can react with amides and amines to form carcinogenic N-nitroso compounds (Sepahvand et al., 2021; van Breda et al., 2021). Benzoates and sorbates are prone to form potential mutagenic compounds (Piper and Piper, 2017). Meanwhile, sulfite-containing preservatives and toxic parabens used with methylisothiazolinone and methylchloroisothiazolinone may trigger allergic reactions (Anand and Sati, 2013). Thereby, bio-preservation and bio-protection have become commercially important in the production of fresh produce, especially those of animal origin (Rathod et al., 2021b).

The incorporation of naturally-derived antimicrobials (e.g., herbs, EOs, and other extracts) into foodstuffs is an effective way to control spoilage, inhibit the proliferation of pathogenic microorganisms (Tajkarimi et al., 2010), and increase consumer acceptance (Yu et al., 2021). EOs from medicinal and aromatic plants have emerged as ideal substitutes for synthetic food additives due to their strong antimicrobial effect and lower health concerns (Busatta et al., 2008; Yu et al., 2021). Nonetheless, the inhibitory effect of these secondary metabolites is highly dependent on their chemical composition (Nazzaro et al., 2013) that is influenced by environmental factors, pedoclimatic conditions, genetic background, harvesting time, and extraction methods (Bendiabdellah et al., 2013; Gu et al., 2019; Sadeh et al., 2019). The distilled oils consist of around 20–60 low molecular weight (usually less than 300 Da) volatile compounds, mainly terpenes, terpenoids, alcohols, phenols, hydrocarbons, aldehydes, and their derivates (Basavegowda and Baek, 2021; Wadhwa et al., 2017). In most cases, the main constituents (20–95%) determine the biological properties of EOs (Shaaban et al., 2012), but other minor and less active components may also contribute to the overall antimicrobial activity (Ghabraie et al., 2016; Lv et al., 2011).

Volatiles rich in phenolics have shown the strongest antimicrobial activity against both gram-positive and gram-negative bacteria (Boskovic et al., 2015) as the hydrophilic part can interact with the polar part of the cell membrane, while the hydrophobic part reacts with the inner part. Thus, important constituents like thymol can easily penetrate and disrupt the cell membrane of microorganisms causing enzyme system impairment, cellular content loss, and, ultimately, cell death (Basavegowda and Baek, 2021; Lambert et al., 2001). However, the EO constituents may have different targets and their antimicrobial efficacy relies on multiple action mechanisms by affecting the respiration, energy metabolism, genetic material, cell wall, or cell membrane (Ju et al., 2018; Kerekes et al., 2015). Therefore, it is difficult to predict the susceptibility of certain strains (Kerekes et al., 2015). Additionally, gram-positive bacteria tend to be more receptive to cell wall-targeting EOs than the gram-negative species that possess an outer membrane (Basavegowda and Baek, 2021; Burt, 2004). The presence of this hydrophilic lipopolysaccharide membrane restricts the traverse of hydrophobic compounds and provides gram-negative bacteria a higher tolerance to EO constituents (Hyldgaard et al., 2012; Tajkarimi et al., 2010). In contrast, the cell wall of gram-positive bacteria is less complex. It contains 90–95% of peptidoglycan that allows the diffusion of hydrophobic compounds (Nazzaro et al., 2013).

Besides the antibacterial activity, EOs and their bioactive constituents can act as fungicidal agents by forming a charge-transfer complex with an electron donor to fungal cells (Bhuyan et al., 2010; Chutia et al., 2009). Nonetheless, some EOs may be able to trigger an adaptive response in fungi (Kerekes et al., 2015) and stimulate their germination. This tolerance might be attributed to the mechanism developed by certain pathogenic species using secondary metabolites as a signal to initiate appressorium formation, germination, and infection (Chutia et al., 2009).

Previous studies have also indicated that plant derivates, such as EOs, can be used as meat preservatives to combat different disease-causing and spoilage microorganisms. Therefore, in the next sections, the *in vitro* and *in vivo* antimicrobial properties of *Thymus vulgaris* EO and thymol will be discussed.

### 2.1 Preservative effect of *Thymus vulgaris* essential oil against foodborne pathogens in meat and meat products

The genus *Thymus* comprises approximately 300–400 species (Fani and Kohanteb, 2017; Mancini et al., 2015). Common thyme (*Thymus vulgaris* L., Lamiaceae) is one of these evergreen aromatic plants, which is grown in various parts of the world for commercial purposes, mainly in the Mediterranean regions of Europe, North Africa, and Asia (Fani and Kohanteb, 2017; Ferreira et al., 2016; Nieto, 2020; Satyal et al., 2016; Silva et al., 2021). Nonetheless, it is collected wild and cultivated in most European countries, with Spain as the top EO-producer (Vouillamoz and Christ, 2020). The plant grows well under temperate to hot, dry, sunny climate conditions, but it can also tolerate coarse, rough soils (Hosseinzadeh et al., 2015). The 10–40 cm high sub-shrubs have reddish stems with short hairs, small, oval, and highly aromatic, greenish-grey leaves with spheroidal glands, and clusters of white, pink, or purple flowers during late spring and early summer (Fani and Kohanteb, 2017; Hemmati et al., 2019; Mewes et al., 2008; Nezhadali et al., 2014; Patil et al., 2021; Pavela et al., 2018; Silva et al., 2021).

Traditionally, the herb has been utilized as a culinary ingredient and medicinal plant to flavor foodstuffs and treat wounds, as well as illnesses such as bronchitis, whooping cough, laryngitis, gastritis, diarrhea, and upper respiratory congestion (Fani and Kohanteb, 2017; Patil et al., 2021; Satyal et al., 2016). However, in recent years, *Thymus vulgaris* has gained popularity and become one of the most studied species in the genera (Patil et al., 2021; Taghouti et al., 2020) as it contains highly promising active ingredients that show strong antimicrobial activity against fungi and bacteria (Oliva et al., 2015). Although thyme extracts are rich sources of flavonols, phenolic acids, flavanones, flavones, flavonoids, saponins, alkaloids, steroids, and tannins (Nieto, 2020), EOs have attracted the most attention due to their biologically active components that have shown antagonistic effect against food originated microbes (Burt et al., 2007; Sepahvand et al., 2021).

The EO of *Thymus vulgaris* is stored in glandular peltate trichomes found on both sides of the leaves. The extraction of oils from the flowering plants (mostly leaves and flowers) is usually performed by different methods including conventional hydro-distillation, steam distillation, steam and water distillation, maceration, expression, or empyreumatic distillation, but new approaches have been also introduced (e.g., microwave-assisted EO extraction, ohmic-extraction, supercritical fluid extraction, pressurized solvent extraction, ultrasound-assisted extraction, etc.) due to the vulnerability and thermal sensitivity of these molecules (Gavahian et al., 2012; Golmakani and Rezaei, 2008; Lucchesi et al., 2004; Tavakolpour et al., 2016; Vouillamoz and Christ, 2020). The main compounds of the obtained EO are terpenoids and phenolic derivates including thymol, carvacrol, α-terpineol, 1,8-cineole, borneol, geraniol, p-cymene, thujanol, γ-terpinene, and caryophyllene (Silva et al., 2021). However, the composition of EOs is highly influenced by intrinsic, ecological, and technological factors and, thus, they can express different effects as biopreservatives (Ed-Dra et al., 2021; Mancini et al., 2015).

Studies such as that conducted by Boskovic et al. (2015), Nezhadali et al. (2014), and Reyes-Jurado et al. (2019) have investigated the *in vitro* efficacy of thyme EO against important foodborne pathogens and other food-related microorganisms. De Carvalho et al. (2015) demonstrated that the minimal inhibitory concentration (MIC) for thyme EO against *Listeria monocytogenes* and *Staphylococcus aureus* was 2.5 μL/mL. Similar results had been reported previously by Rota et al. (2008), who showed that the EO of *Thymus vulgaris* (thymol chemotype) inhibited the growth of various gram-positive and gram-negative species including *Salmonella* Enteritidis, *Salmonella* Typhimurium, *Escherichia coli* O157:H7, *Escherichia coli*, *Listeria monocytogenes*, *Yersinia enterocolitica*, *Shigella flexneri*, *Shigella sonnei*, and *Staphylococcus aureus* with inhibition zones ranging from 19.6 to 45.0 mm. Thyme EO containing larger amounts of thymol, *p*-cymene, and linalool also exhibited strong antibacterial activity against seven isolated *Enterobacteriaceae* strains (Benameur et al., 2019). In the same vein, Ed-Dra et al. (2021) indicated that the half-MIC concentration (0.25%) of this volatile liquid caused sub-lethal damage to different serotypes of *Salmonella enterica* subsp*. enterica*. Al-Bayati (2008) found that thyme EO was active against all the tested pathogens (e.g., *Staphylococcus aureus*, *Bacillus cereus*, *Proteus vulgaris*, etc.) except *Pseudomonas aeruginosa*. Meanwhile, Kačániová et al. (2012) reported that various concentrations of thyme EO (0.750 and 0.375 mL/mL) possessed strong antibacterial activity against *Escherichia coli* and *Bacillus cereus*.

Additionally, a number of studies have investigated the inhibitory effect of thyme EO on biofilm formation. Kang et al. (2018) showed that the inhibition rate of *Thymus vulgaris* EO (0.5 mg/mL) on *Bacillus cereus* biofilm formation was 80.79%. These results were supported by Desai et al. (2012) who demonstrated that 24-hour long contact with thyme EO (0.25–0.5%) reduced the formation of *Listeria monocytogenes* biofilms on the surface of stainless steel coupons and inactivated the bacterial cells completely. Furthermore, Liu et al. (2020) found that thyme oil can inhibit the transcription of *ebp* and *epa* gene clusters and, consequently, reduce cell adherence as well as inhibit the exopolysaccharide synthesis in *Enterococcus faecalis* biofilms.

To confirm the antifungal activity of *Thymus vulgaris*, Klarić et al. (2006) compared the inhibitory effect of thyme EO with thymol on *Absidia, Alternaria, Aspergillus, Chaetomium, Cladosporium, Mucor, Penicillium, Rhizopus, Stachybotrys, Trichoderma,* and *Ulocladium* spp. The used oil showed a strong antagonistic effect (MIC: 3.2–50.2 μg/mL) against these fungi. In addition, Oliveira et al. (2020) reported that thyme EO can also have anti-aflatoxigenic effects: the lowest concentration of this volatile (0.1 μL/mL) reduced the aflatoxin B_1_ production of *Aspergillus flavus* by 80% compared to the control treatment without thyme EO. Overall, their molecular analysis proved that the EO in a concentration of 0.25 μL/mL downregulated all the tested genes (lipA, meT, and laeA) and, as a result, affected the establishment and development of the fungi too.

To demonstrate the effectiveness of the volatile oil under *in vivo* conditions, Aureli et al. (1992) added thyme EO to minced pork meat infected with *Listeria monocytogenes*. The results of this experiment showed that the application of EO gradually reduced the number of viable *Listeria monocytogenes* by circa 2 logs over the first week of storage. Additionally, Pesavento et al. (2015) used EO-supplemented beef meatballs to verify the antibacterial activity of volatiles in meat products. They found that the thyme oil at a concentration of 0.5% was bacteriostatic in its action against *Listeria monocytogenes* at 4 °C, while the 1% and 2% concentrations gradually decreased the microbial load of the samples. Barbosa et al. (2009) demonstrated that thyme EO added to irradiated minced meat had a bacteriostatic effect on *Staphylococcus aureus*, *Listeria monocytogenes*, *Escherichia coli*, and *Salmonella* Enteriditis at 5 °C. Similarly, Amariei et al. (2016) observed that rosemary (*Rosmarinus officinalis* L.), thyme, and oregano (*Origanum vulgare* L.) EOs (0.5–1.5%) had the same effect on the microbiological stability of minced meat. By the end of the 3rd day, decreased LAB, yeast, and mold counts were recorded in the supplemented samples compared to the control. Consequently, the added EOs improved the physicochemical properties of the minced meat and prolonged its shelf-life. Huang et al. (2021) found that the application of thyme EO amendment inhibited the growth of members of the *Enterobacteriaceae* family in Chinese smoked horsemeat sausages, while the number of LABs did not differ remarkably between the treatments. In addition to the microbiological analysis, Lucera et al. (2009) proved that the augmentation of air-packed poultry patties with plant-based bioactive compounds (i.e., thymol and carvacrol) as individual antioxidants retarded the lipid oxidation process and maintained a more stable color than the control sample.

### 2.2 Preservative effect of thymol against foodborne pathogens in meat and meat products

Based on the EO composition, six major chemotypes of *Thymus vulgaris* can be distinguished from each other (Galovičová et al., 2021; Silva et al., 2021). Thymol is one of the dominant constituents in thyme EO (up to 40–80%) (Nieto, 2020; Satyal et al., 2016) that contributes to its antimicrobial and antioxidant activities (Gonçalves et al., 2017). Therefore, this monoterpene derivate of cymene has been sought to be utilized as antiseptic, antioxidant, and antimicrobial agent for food and medicinal purposes (Jafri and Ahmad, 2020; Salehi et al., 2018; Silva et al., 2021).

The studies presented thus far provide evidence that thymol, as a natural biocide, is able to disintegrate the outer membrane of microbial cells, enhance the lipopolysaccharide release and 1-N-phenylnaphthylamine uptake, induce changes in fatty acid composition and phospholipids, influence the synthesis of genetic material, permeabilize and depolarize the cytoplasmic membrane, and cause the leakage of potassium ions, protons, and ATP leading to the loss of membrane potential and the impairment of energy metabolism (Di Pasqua et al., 2010; Helander et al., 1998; Xu et al., 2008). In accordance with these, Trombetta et al. (2005) showed that thymol exerted toxic activity against *Staphylococcus aureus* and *Escherichia coli* that may be due to the perturbation of the lipid bilayer of plasma membranes, the alteration of cell membrane integrity, and the release of intracellular materials. Similarly, Wang et al. (2017) investigated the antibacterial efficacy of thymol against *Staphylococcus aureus.* The addition of this phenolic monoterpene induced alterations in the fatty acid composition of the membrane, and consequently, disrupted cell membrane integrity. Moreover, thymol was able to bind to the genomic DNA of *Staphylococcus aureus* and made DNA molecules aggregated.

### 2.3 Considerations for using thyme essential oil and thymol in meat commodities

One of the most important considerations of using EOs and their bioactive constituents as bio-preservatives is the legal dose (Gavahian et al., 2020) that can be added to meat commodities. Fortunately, both thyme EO and the source plant (*Thymus vulgaris*) are generally recognized as safe (GRAS) by the Food and Drug Administration/FDA (21 CFR 182.10, 21 CFR 182.20) (Federal Register, 2009). The herb itself is a low-cost commodity, which has shown substantial antibacterial, antifungal, and antiviral activity (El-Sayed and Youssef, 2019; Kowalczyk et al., 2020). Nonetheless, applicability of herbs and spices as a natural food preservative is limited because of their chemical variability (Martínez-Graciá et al., 2015). Similarly to thyme and its EO, thymol is FDA approved when it is added to the food as a synthetic food flavoring agent (21 CFR 172.515), preservative, or indirect food additive of adhesives (21 CFR 175.105) (Federal Register, 2009). Since thymol has low levels of associated risks at normal ingestion levels (Liu et al., 2021; Rathod et al., 2021a), it also has been registered for food application by Regulation (EC) No 1334/2008 and Regulation (EU) No 872/2012 (European Commission, 2012; Pinto et al., 2021; Rathod et al., 2021a). Nonetheless, the use of these antimicrobials in food matrices needs to be regulated by authorities more vigorously by defining the targets, effective range, minimal inhibitory concentration, and the safety data (allergenicity, toxicity) as well (Tajkarimi et al., 2010).

Another important aspect to consider while using plant-based antimicrobials as meat preservatives is the sustainability and environmental impact of EO production and application. Since the EO content of thyme leaves is relatively low (around 0.8–2.6%), a large amount of waste is generated during the distillation process (Gavarić et al., 2015). Traditionally, the remaining biomass is treated by incineration or landfilling causing serious environmental problems worldwide (Greff et al., 2021). However, the solid plant waste, as well as the hydrolat contains exploitable bioactive compounds that can be recovered and utilized as natural preservatives in foodstuffs. Along with the antimicrobial effect, the addition of aromatic plant waste could contribute to the functionality of products by increasing the total dietary fiber and polyphenol content (Pogačar et al., 2016; Popa et al., 2021; Vasileva et al., 2018).

## 3 Limitations of thyme essential oil and thymol as bio-preservatives in meat products and potential solutions

Although both thyme EO and thymol have the potential to be used as bio-preservatives for the production of safe meat commodities, their practical application is still hampered by their unstable nature, volatility, thermosensitivity, low water solubility, lower efficacy caused by the interactions with food matrix components, and the diverse microbial composition of raw meat (Falcone et al., 2007; Liu and Liu, 2020; Mastromatteo et al., 2009; Perez et al., 2019; Ren et al., 2021; Sepahvand et al., 2021).

Accordingly, former studies have indicated that thyme EO and thymol had no or only mild antimicrobial effect under *in vivo* conditions. For example, Solomakos et al. (2008) found that the supplementation of 0.6% (v/w) EO showed no inhibitory activity against *Escherichia coli* O157:H7 strains in minced beef meat at 4 °C. However, the antimicrobial potential of this EO was temperature-dependent as the populations of the tested pathogens were significantly lower (∼4.8 log CFU/g) at 10 °C than in the control (∼7.1 log CFU/g). Similarly, Gouveia et al. (2016) found that thyme EO mixed to *sous vide* cook-chill beef samples at MIC level (0.39 v/v) did not alter the density of *Listeria monocytogenes* significantly at 2 °C or 8 °C compared to the EO-free sample. In such a situation, to achieve the same inactivation level as under *in vitro* conditions, higher doses of EOs may be required in real food matrices. Nonetheless, such concentrations often interfere with the sensory characteristics of food products causing unpleasant changes in color and flavor (Chien et al., 2016; Radünz et al., 2020).

The efficacy of these plant-based compounds can be easily improved in combination with other hurdles including high-hydrostatic pressure, low dose irradiation, low temperature, other active components, and modified atmosphere packaging (MAP) (Blázquez et al., 2018; Lucera et al., 2009; Mastromatteo et al., 2010b; Schirmer and Langsrud, 2010). The hurdle technology is a mild preserving technique that can attenuate the growth of pathogenic and spoilage microorganisms without imposing adverse effects on meat quality. Due to the synergy among selected hurdles, less quantity of the used bio-preservatives may be required (Ishaq et al., 2021; Mastromatteo et al., 2009, 2011) to ensure the microbiological safety of fresh meat and meat commodities. For this reason, the negative effects of thyme EO and thymol on food quality can be significantly reduced.

### 3.1 Combined application of antimicrobials

One possible solution for the aforementioned problems is the combined application of antimicrobials. Gutierrez et al. (2008) reported that the simultaneous use of *Thymus vulgaris* and *Origanum vulgare* EOs had an additive effect against *Bacillus cereus* and *Pseudomonas aeruginosa*. Thyme EO combined with basil (*Ocimum basilicum L.*) or sage (*Salvia triloba* L.) oil showed a similar effect against *Listeria monocytogenes*. Mohammadpourfard et al. (2020) evaluated the effectiveness of thymol in combination with astaxanthin and nitrite. The results showed that the optimal formula (thymol 125 ppm, astaxanthin, 450 ppm, nitrite 120 ppm) inhibited the growth of *Clostridium perfringens* and provided better organoleptic properties to common and probiotic cooked sausages. Lu and Wu (2012) developed two thymol-based washing solutions to sanitize chicken breast samples. Compared to the chlorine solution, both treatments (thymol with acetic acid and thymol with acetic acid and sodium dodecyl sulfate) achieved similar log reduction on *Salmonella enterica* Kentucky without significantly affecting the pH or the texture values of the meat samples. Moon et al. (2020) used a commercially available bacteriophage with thymol (0.8% and 1.6%) to control the growth of *Salmonella* in fresh chicken meat. The sequential application of these treatments (2.0–2.9 log reduction on day 5) was remarkably better than the single treatments (0.8–1.8 log reduction on day 5). Klupsaite et al. (2020) prepared *Ile de France* and *Suffolk* breed lamb meat samples supplemented with *Lactobacillus plantarum* LUHS135 and/or *Thymus vulgaris* EO (0.1% v/v). Overall, the microbiological composition (total enterobacteria count, mold and yeast count) of the meat treated with the combination of LUHS135 and thyme EO was improved compared with the single EO treatment. According to Hastaoğlu et al. (2021), the combined application of thymol with nitrite and beet root extract also provided high antimicrobial (LAB and total aerobic psychrophilic bacteria) and antioxidant effects with elevated total phenolic content in beef Mortadella. However, rosemary oil was more suitable for the production of carmine-free and reduced nitrate meat commodities due to its sensorial effect. Similarly, Lages et al. (2021) found that the use of thyme EO with powdered beet juice has the potential to substitute nitrates and nitrites in meat sausages.

In conclusion, these studies have outlined that thyme oil and thymol may be even more effective in combination with other antimicrobials, suggesting a synergistic and additive effect.

### 3.2 Marinades infused with bioactive compounds

Marination is a meat preservation technique during which a water-oil emulsion typically containing sugar, salt, acids, spices, rheology-improving additives, aroma strengtheners, and antimicrobial agents is added to the meat to improve its shelf life, taste, and consistency (Björkroth, 2005). Although marinades may be characterized by antimicrobial potential due to the high concentration of salt, preservatives, and spices, and acidic pH, they may be less effective under real processing conditions. Therefore, EOs may be used during marinade formulation to improve the bactericidal effect (Moon et al., 2017). Moon and Rhee (2016) reported that the combination of soy sauce with thymol increased its antibacterial activity against both gram-positive and gram-negative pathogens without significantly changing the natural flavor of the sauce. Thanissery and Smith (2014) added thyme and orange EOs to a marinade solution at a concentration of 0.5%. As a result of the dip application of this mixture, *Salmonella* Enteritidis and *Campylobacter coli* counts were remarkably decreased on both skinless broiler breast fillets and whole wings compared to the marinated and non-marinated control samples. Karam et al. (2020) demonstrated that the addition of active EO components (0.4% and 0.8% w/w) elongated the microbial shelf-life of marinated beef samples stored in air or vacuum packaging by 3–6 days. Although the marinades with high concentrations of carvacrol and thymol (0.8%) were more effective in controlling the growth of the spoilage microflora (*Pseudomonas* spp., *Brochothrix thermospicata*, LAB, yeasts, molds) and indicator microorganisms, the treatments containing low concentrations of plant-based antimicrobials (0.4%) were more preferred due to their lower sensory impact. By applying a cold marinating method, Moon et al. (2017) found that teriyaki sauce augmented with thymol (0.5%) inhibited the growth of indigenous bacteria and effectively inactivated *Salmonella* Typhimurium, *Listeria monocytogenes*, and *Escherichia coli* O157:H7 in inoculated beef slices. Similarly, Nisiotou et al. (2013) used thyme EO to improve the antimicrobial effect of wine-based marinades and reported that the immersion of fillets into EO-containing marinade reduced the populations of acid-adapted and non-adapted *Salmonella* Typhimurium strains more rapidly during the 19-day long storage at 5 °C compared to the sample marinated in wine. On the other hand, Schirmer and Langsrud (2010) claimed that thymol alone or in combination with other active ingredients (e.g., citric acid, rosemary extract, allyl isothiocyanate, and grape fruit seed extract) had no antibacterial effect in marinated pork.

### 3.3 Encapsulation of bioactive compounds

The encapsulation process of EOs and oil constituents (e.g., molecular inclusion complexation with host molecules, encapsulation into liposomes and micelles, coacervation with various proteins, carbohydrates, and polymers) also stands out as a feasible, commercially available, and rapidly expanding technology that prevents the evaporation as well as oxidation of these compounds, and masks the unwanted sensory changes in meat products without diminishing their effects (Cui et al., 2017; Ghaderi-Ghahfarokhi et al., 2016; Liu et al., 2010; Radünz et al., 2020; Turasan et al., 2015; Wattanasatcha et al., 2012). During the process, a physical barrier is formed between the surroundings and the core material (Gonçalves et al., 2017; Plati and Paraskevopoulou, 2022) that controls the release of encapsulated ingredients into the food matrix and, consequently, improves the biological effects compared to their non-encapsulated counterparts (Barros et al., 2022). This is in agreement with the results of Ghaderi-Ghahfarokhi et al. (2016) who found that thyme EO encapsulated by chitosan nanoparticles had a greater influence on the inhibition of *Enterobacteriaceae*, *Staphylococcus aureus*, LAB, yeasts, and molds than the free EO. Likewise, Radünz et al. (2020) prepared casein-maltodextrin encapsulated thyme EO to improve the microbiological properties of hamburger-like meat products. Criado et al. (2019) investigated the antilisterial activity of *Thymus* oil-loaded alginate beads (1–3%) in irradiated (0–3.0 kG y) ground meat. Overall, a synergistic effect of gamma irradiation with EO-containing alginate beads was observed against *Listeria innocua* during the 14-day long storage. Wattanasatcha et al. (2012) used thymol-loaded ethylcellulose-methylcellulose particles to inhibit the growth of *Staphylococcus aureus*, *Pseudomonas aeruginosa*, and *Escherichia coli*. In the case of *Staphylococcus aureus* and *Escherichia coli,* the MIC and the minimal bactericidal concentration (MBC) values of free and encapsulated thymol were similar (0.01–0.313 mg/ml), while the encapsulated thymol was less effective against *Pseudomonas aeruginosa*.

The use of liposome-based systems is also a promising and well-established technology as these enclosed spherical microscopic vesicles are low toxicity, biocompatible, biodegradable, and non-immunogenic (Cui et al., 2017; Hammoud et al., 2019). Moreover, the encapsulation of thymol into lipoid S100-liposomes improved the chemical stability and solubility of this bioactive compound (Hammoud et al., 2019).

### 3.4 High-pressure processing

High-pressure processing (HPP) is one of the most popular non-thermal preservation technologies that can be combined with biopreservation methods to achieve a stronger antimicrobial effect without affecting the nutritional value and organoleptic properties of certain products (Chien et al., 2019; Pérez-Baltar et al., 2019). For example, Chien et al. (2016) found that the simultaneous use of HPP and thymol reduced the damaging effect of the individual treatments on the food quality. Furthermore, Aras et al. (2020) investigated the efficiency of pressure-based treatments in combination with thymol and mild heat. The results showed that the treatment with 0.15% of thymol at 4 and 40 °C had no inhibitory effect on the growth of *Escherichia coli* O157:H7 or the mesophilic background microbiota of meat homogenate. However, the combination of thymol and elevated hydrostatic pressure (400 MPa) with mild heat treatment (40 °C) eliminated an appreciable amount of these microorganisms (3.1 and 4.4 log CFU/ml) after 9-minute long treatment. In addition, Pérez-Baltar et al. (2019) combined HPP (450 MPa) with natural antimicrobials to treat dry-cured ham samples contaminated with *Listeria monocytogenes*. The use of thymol (1.25 mg/g) showed a synergistic antibacterial activity during the 30-day storage at 4 and 12 °C by reducing *Listeria monocytogenes* levels of the final products by 0.7–1.0 log units compared to the single treatments.

### 3.5 Modified atmosphere packaging (MAP)

During the last decades, various food packaging methods and materials have been proposed and developed to decrease the spoilage rate of meat products. MAP is a widely used technique to supplement the low-temperature preservation of fresh and cooked meat (Chen et al., 2021; Mastromatteo et al., 2010a; Xiong et al., 2020), but it may not be sufficiently efficient in the case of certain foodstuffs. The combination of MAP methods with natural antimicrobial compounds, however, may improve the physicochemical and microbiological shelf life of these products (Mastromatteo et al., 2010a).

For this reason, Mastromatteo et al. (2011) investigated the effect of MAP (20% CO_2_, 5% O_2_, 75% N_2_) supplemented with thymol on the shelf life of reduced pork back-fat content sausages. Based on the results of their microbiological analysis, this combination had an additive effect regarding the total viable count (1.97 log reduction) and the populations of psychrotrophic bacteria (1.82 log reduction), coccus-shaped LAB (1.85 log reduction), and *Enterobacteriaceae* (0.69 log reduction). Overall, the shelf life value of the product was more than five days. Karabagias et al. (2011) showed that the use of thyme EOs (0.1%) with MAP (80% CO_2_, 20% N_2_) extended the shelf life of fresh lamb meat by 14–15 days at 4 °C. Similar results were obtained by D'Amato et al. (2016): the combined application of MAP and *Thymus vulgaris* EO (20 mg/ml) improved the shelf life of fresh pork meat.

### 3.6 Active packaging

Along with MAP, the incorporation of antimicrobial agents into biopolymers has been receiving increasing attention due to biodegradability and reduced environmental impact (Reis et al., 2022; Tawakkal et al., 2016).

Tornuk et al. (2015) prepared linear low-density polyethylene matrix-based nanocomposite films with active nanoclays loaded with thymol. These packaging materials suppressed the proliferation of *Escherichia coli* O157:H7 in fresh beef (0.52–0.92 log decrease). Moreover, the incorporated thymol reduced the populations of total mesophilic aerobic bacteria, LAB, as well as total yeast and mold of vacuum-packed sucuk significantly without affecting the thiobarbituric acid values. Petchwattana and Naknaen (2015) developed a polybutylene succinate/thymol film that was tougher and softer due to the plasticization effect of thymol. The biodegradable packagingcontaining 10 wt% of thymol effectively inhibited the growth of *Staphylococcus aureus* and *Escherichia coli*. All things considered this polymer film was effective over 15 days, offering a great alternative for short-cycle food packaging. Tawakkal et al. (2016) applied polylactic acid (PLA) film incorporated with kenaf fibers and/or thymol to control the growth of *Escherichia coli* inoculated on the surface of processed sliced chicken samples. During the direct contact test, PLA/kenaf/thymol formulation containing higher thymol content (30 % w/w) showed strong antimicrobial activity and reduced the number of viable *Escherichia coli* under the detection limit by the 19^th^ day of the experiment. Meanwhile the PLA films supplemented with thymol only were less effective against this gram-negative bacteria. In addition, Guarda et al. (2011) found that microencapsulated thymol and carvacrol inhibited the growth of potential pathogens; however, only the antimicrobial biofilm containing 10% of thymol and 10% of carvacrol showed a weak inhibitory activity against *Listeria innocua*, *Escherichia coli*, and *Staphylococcus aureus* (IZ: 8.8–11.3 mm).

In the case of edible coatings containing bioactive compounds, the packaging material can be directly applied to the surface of food products to improve the sensorial, antioxidant, and antimicrobial properties (Reis et al., 2022). Gedikoğlu (2022) used pectin-based edible coating with *Thymus vulgaris* and *Thymbra spicata* EOs to improve the shelf life of sliced bolognas. Although thymus oil has potent antimicrobial properties *in vitro*, the edible coating supplemented with the EO blend was effective against *Salmonella typhimurium* only and did not affect the growth of *Listeria monocytogenes* and *Staphylococcus aureus*. Nonetheless, the applied material elongated the shelf life of meat products by controlling the growth of spoilage microorganisms (LAB, yeasts, and molds). Wang et al. (2022a) used gelatin/zein nanofiber films loaded with thymol to decelerate the spoilage of chilled chicken breast. At the end of the 12-day storage period, this edible coating reduced the total viable bacterial counts notably to 6.61 logCFU/g. To overcome the instability and low solubility of thyme oil in water-based products, nanoemulsion techniques have also been implemented. The formed nanostructures (20–200 nm) in EO-based nanoemulsions can improve the antimicrobial activity by encouraging inert cellular absorption devices and increasing the total surface area of nano-scaled particles (El-Sayed and El-Sayed, 2021; Mahato et al., 2019; Moazeni et al., 2021). Liu and Liu (2020) demonstrated that the application of thymol and thyme EO loaded chitosan nanoemulsions controlled the growth of the natural microflora and, consequently, extended the shelf-life of refrigerated pork over 6 days compared to the control sample. These findings are consistent with the results reported by Wang et al. (2022b).

## 4 Conclusion and future perspectives

The use of phytochemicals derived from aromatic and medicinal plants may provide an alternative bio-based preservation method to overcome the negative effects of synthetic food additives. The EO of *Thymus vulgaris* L. and plant-based thymol are characterized by strong antimicrobial potential and can be utilized as bio-preservative agents in meat products. This review paper has summarized the major effects of these phytochemicals on important pathogenic bacteria and fungi. All things considered, most of the *in vitro* and *in vivo* studies confirmed that the use of thyme oil and thymol is an effective way to improve the microbiological safety of fresh meat and meat commodities, but their practical application is still limited due to their unstable nature and strong organoleptic properties. Therefore, further research needs to be conducted to find economically and environmentally more feasible options that can fulfill the requirements of consumers regarding composition, taste, and odor. The latest advances in hurdle technology, active packaging, and encapsulation are also discussed to help improve the development of new bio-preservation methods and increase consumer acceptance.

## Declarations

### Author contribution statement

All authors listed have significantly contributed to the development and the writing of this article.

### Funding statement

Mr. Miklós Posgay was supported by Innovative Scientific Institutions in Domestic Agricultural Higher Education [EFOP-3.6.3-VEKOP-16-2017-00008]. This work was supported by the ÚNKP-22-4-II-SZE-24, ÚNKP-22-3-II-SZE-23 New National Excellence Program of the Ministry for Culture and Innovation from the source of the National Research, Development and Innovation Fund.

### Data availability statement

No data was used for the research described in the article.

### Declaration of interest’s statement:

The authors declare no conflict of interest.

### Additional information

No additional information is available for this paper.
